# ETS1–HMGA2 Axis Promotes Human Limbal Epithelial Stem Cell Proliferation

**DOI:** 10.1167/iovs.64.1.12

**Published:** 2023-01-18

**Authors:** Bofeng Wang, Huizhen Guo, Dongmei Liu, Siqi Wu, Jiafeng Liu, Xihong Lan, Huaxing Huang, Fengjiao An, Jin Zhu, Jianping Ji, Li Wang, Hong Ouyang, Mingsen Li

**Affiliations:** 1 State Key Laboratory of Ophthalmology, Zhongshan Ophthalmic Center, Sun Yat-sen University, Guangdong Provincial Key Laboratory of Ophthalmology and Visual Science, Guangzhou, China.

**Keywords:** limbal epithelial stem cells, ETS1, HMGA2

## Abstract

**Purpose:**

This study aimed to investigate the role and molecular mechanism of ETS1 in the proliferation and differentiation of human limbal epithelial stem cells (LESCs).

**Methods:**

RNA-seq and quantitative real-time PCR were used to determine gene expression changes when *ETS1* and *HMGA2* was knocked down using short-hairpin RNAs or overexpressed by lentivirus. Immunofluorescence and flow cytometry experiments were performed to assess the roles of ETS1 and HMGA2 in LESC proliferation. ETS1-bound *cis*-regulatory elements and target genes in LESCs were identified using chromatin immunoprecipitation sequencing. The epigenetic features of ETS1-binding sites were assessed by the published histone modification and chromatin accessibility profiles.

**Results:**

ETS1 was robustly expressed in LESCs but dramatically reduced on differentiation into corneal epithelial cells (CECs). *ETS1* knockdown in LESCs inhibited cellular proliferation and activated CEC markers (*KRT3*, *KRT12*, *CLU*, and *ALDH3A1*). When *ETS1* was overexpressed during CEC differentiation, LESC-associated genes were upregulated while CEC-associated genes were downregulated. The genome-wide binding profile of ETS1 was identified in LESCs. ETS1 occupied H3K4me3-marked promoters and H3K27ac/H3K4me1-marked enhancers. ETS1-binding sites were also enriched for chromatin accessibility signal. HMGA2 showed a consistent expression pattern with ETS1. ETS1 activates *HMAG2* by binding to its promoter. Knockdown and overexpression experiments suggested that HMGA2 can promote LESC proliferation and inhibits its differentiation.

**Conclusions:**

ETS1 promotes LESC proliferation and inhibits its differentiation via activating HMGA2.

The corneal epithelium is the outermost barrier of corneal tissue, and its structural integrity is crucial for clear vision. Corneal epithelial homeostasis is established and maintained by limbal epithelial stem cells (LESCs) residing in the basal layer of the limbal epithelium.^1^^–^^3^ LESCs can proliferate, migrate, and differentiate into mature corneal epithelial cells (CECs) that replace dead or damaged cells during homeostasis and regeneration, which is required for corneal transparency and normal vision.^1^^–^^5^ Limbal stem cell deficiency, generally caused by mechanical, chemical, or pathological damage, can lead to persistent corneal epithelial disorders and vision loss.^6^^,^^7^ Clinically, the loss or dysfunction of LESCs are associated with multiple pathological changes, such as opacified keratinized epithelium, conjunctivalization, neovascularization, and ulceration.^8^^,^^9^ Recent evidence suggests that transplanting autologous LESCs that were expanded in vitro is a safe and effective approach for treating limbal stem cell deficiency.^10^ Despite the importance of LESCs, the molecular mechanism underlying their functions remain largely unknown.

Transcription factors play key roles in cell fate, identity, and function maintenance.^11^ As a downstream effector of the Ras/MAPK pathway,^12^^,^^13^ the transcription factor ETS1 is only expressed in the proliferative layer of stratified skin epithelium tissue, and it is also expressed robustly in squamous cell carcinomas.^14^^,^^15^ Emerging evidence has demonstrated that ETS1 can repress terminal differentiation of skin keratinocytes and promote tumor cell migration.^16^^–^^19^ However, the functions of ETS1 in LESCs remain unclear. In this study, we found that ETS1 promoted LESC proliferation and inhibited its differentiation by activating HMGA2. ETS1 regulated the expression of downstream genes by occupying the promoters or enhancers. These findings provided novel insights into the regulatory mechanism underlying the balance between proliferation and differentiation of LESCs.

## Material and Methods

### Normal Human Limbus Tissue Samples

All normal human limbus samples were obtained from the Eye Bank of Guangzhou City, Zhongshan Ophthalmic Center (Guangdong, China). This study was approved by the Ethics Committee of Zhongshan Ophthalmic Center of Sun Yat-sen University.

### Isolation and Culture of Human LESCs

Limbus tissues were obtained from postmortem human eyeballs, washed with cold PBS, and cut into small pieces. Subsequently, the limbus pieces were incubated with collagenase IV (17104019; Gibco, Thermo Fisher Scientific, Waltham, MA, USA) at 37°C for 2 h and then with 0.25% trypsin-EDTA (25200072; Thermo Fisher Scientific) for another 15 minutes. Next, the digested tissue pieces were seeded onto Matrigel-coated (BD Bioscience, Franklin Lakes, NJ, USA) polystyrene plates (Corning Inc., Corning, NY, USA). The components of the LESC culture medium were described previously.^20^ Briefly, the culture medium contained DMEM/F12 and DMEM (1:1) with 10% fetal bovine serum (Gibco), 1% penicillin–streptomycin (15140122; Thermo Fisher Scientific), 5 mg/mL insulin (I5500; Sigma-Aldrich Corp., St. Louis, MO, USA), 10 ng/mL EGF (GF144; Millipore, Burlington, MA, USA), 0.4 ug/mL hydrocortisone (386698; Millipore), 10^−10^ M cholera toxin (C8052; Sigma-Aldrich Corp.) and 2 nM 3,3ʹ,5-triiodo-L-thyronine (T2877; Sigma-Aldrich Corp.).

### Immunofluorescence Staining

Before dehydration and paraffin embedding, normal human limbus samples were fixed in 10% neutral-buffered formalin for one hour. Deparaffinization was performed before staining. Cell samples were fixed with 4% paraformaldehyde at room temperature for 15 minutes. Next, the tissues or cell samples were incubated with a PBS solution containing Triton X-100 and 3% BSA at room temperature for one hour. Subsequently, the samples were incubated with primary antibodies at 4°C overnight, followed by incubation with secondary antibodies for one hour and Hoechst 33258 dye (Thermo Fisher Scientific) for 15 minutes at room temperature. All images were obtained using a Zeiss LSM 800 microscope (Zeiss, Oberkochen, Germany). Antibodies are listed in Supplementary Table S1.

### In Vitro differentiation of LESCs

LESCs were seeded and grown to 100% confluence in LESC medium. The medium was then changed to a complete keratinocyte serum-free medium (KSFM; Thermo Fisher Scientific) with 120 µM calcium chloride, in which the cells were cultured for up to one week. The differentiation medium was changed every day. The differentiated cells were identified by qPCR analysis and immunofluorescence staining of CEC markers.

### Cell Proliferation Assay

The 5-ethynyl-2ʹ-deoxyuridine (EdU) Cell Proliferation Kit (C0071S; Beyotime Institute of Biotechnology, Jiangsu, China) was used to measure the proliferative capacities of cells. Cells were treated with EdU for two hours. Fixation, detergent, permeabilization, and EdU staining were performed according to the manufacturer's protocol. Cell-ID 5-(and 6)-carboxyfluorescein diacetate succinimidyl ester (CFSE) Cell Proliferation Kit (A001; ABP Bioscience, Beltsville, MD, USA) was also used to examine cell proliferation. The cells were labeled with 3 µM CFSE for 20 minutes, and the labeling solution was replaced with fresh prewarmed LESC medium. After culturing the cells for three days, the CFSE signal intensity was detected by flow cytometry according to the manufacturer's protocol.

### Gene Knockdown and Overexpression

Short-hairpin RNAs (shRNAs) targeting *ETS1* or *HMGA2* were designed from the Merck online tool and subcloned into the PLKO.1 plasmid. A scrambled shRNA that did not target any known gene was used as a negative control. For ETS1 and HMGA2 overexpression, the coding sequences of *ETS1* or *HMGA2* were inserted into the PCDH-CMV plasmid. For lentivirus package, the target plasmid and packaging plasmids psPAX2 and pMD2.G were co-transfected into HEK293T cells. Lentivirus particles were collected for two days post-transfection. For lentiviral infection, the cells were infected for 24 hours with lentiviral particles in fresh LESC medium containing 8 µg/mL polybrene. Positive cells were selected by incubating the cells in a medium containing 2 µg/mL puromycin for two days after transfection. shRNAs targeting *ETS1* and *HMGA2* are listed in Supplementary Table S2.

### Quantitative Real-Time PCR (qPCR)

Total RNAs were extracted using the RNeasy Mini Kit (74106; Qiagen, Hilden, Germany) according to the manufacturer's instructions and reverse-transcribed to cDNA using the PrimeScript RT Master Mix Kit (HRR036A; Takara Biotechnology Co., Kyoto, Japan). qPCR was performed using an iTaq Universal SYBR Green Supermix Kit (1708880; Bio-Rad Life Science, Hercules, CA, USA).

### RNA-seq Analysis

For cDNA library construction, the sheared RNAs were reverse transcribed using the NEBNext RNA First- and Second-Strand Synthesis Module (New England Biolabs, Ipswich, MA, USA). The KAPA Library Preparation Kit (Kapa Biosystems, Wilmington, MA, USA) was used for end repair, A-tailing, adapter ligation, and amplification. DNA libraries were sequenced on an Illumina NovaSeq 6000 instrument with paired-end 150 reads setting. To calculate read counts for each gene, the trimmed reads were aligned to the human hg19 reference genome using STAR software (version 2.6.1a).^21^ The RSEM tool (version 1.3.0)^22^ was used to generate transcripts per kilobase million values representing the gene expression levels. Significantly differentially expressed genes were determined using DESeq2 (version 1.20.0),^23^ with a fold change ≥2 and a *P* value < 0.05 as thresholds. Gene Ontology (GO) biological process enrichment analysis was conducted using the clusterProfiler R package (version 3.18.1),^24^ with a *P* value cutoff of 0.05 and a *q* value cut-off of 0.05.

### Chromatin Immunoprecipitation Sequencing (ChIP-seq) Analysis

The ChIP-seq protocol used in this study was based on our previous research.^20^^,^^25^^,^^26^ Briefly, cells were fixed in 1% formaldehyde at room temperature for 10 minutes, and the crosslinked chromatin was sheared to obtain 300 to 500 bp DNA fragments using a Covaris M220 focused-ultrasonicator in sonication buffer (50 mM HEPES-NaOH, pH 7.5, 500 mM NaCl, 1 mM EDTA, 0.1% Na-deoxycholate, 1% TritonX-100, and 0.1% SDS). The DNA fragments were incubated with primary antibodies (anti-ETS1, CST, Cat no. 14069) at 4°C overnight and then with Protein A/G Dynabeads (Invitrogen) for one hour. The beads were washed successively in high-salt buffer, low-salt buffer, and TE buffer. After elution from the beads and de-crosslinking, the DNA fragments were purified using a MinElute PCR Purification Kit (Qiagen). Finally, the purified DNA was used to construct DNA libraries with the KAPA Hyper Prep Kit (KK8502; Kapa Biosystems), which were sequenced using an Illumina NovaSeq 6000 instrument.

For ChIP-seq data, reads were trimmed and aligned to the human hg19 reference genome using Trimmomatic tool^27^ and BWA software,^28^ respectively. The Picard MarkDuplicates tool was used to select unique reads for downstream analysis. MACS2^29^ was used for peak calling. The HOMER mergePeaks command was used to generate overlapping peaks between two biological replicates. The deepTools multiBamSummary tool was used for Pearson's correlation coefficient analysis. Motif enrichment was performed using HOMER findMotifsGenome.pl.

The ChIP-seq data for histone modifications and ATAC-seq data were obtained from Gene Expression Omnibus under the accession number: GSE156273. The TP63 ChIP-seq data were obtained from Gene Expression Omnibus under the accession number: GSE192625.

### Statistical Analysis

Student's *t*-test was performed using GraphPad Prism 6. All results are presented as the mean ± standard error (SE). Statistically significant data are indicated by asterisks (*P < 0.05, **P < 0.01, *** *P* < 0.001).

## Results

### ETS1 was Specifically Expressed in The Limbal Epithelium

**Figure 1.**
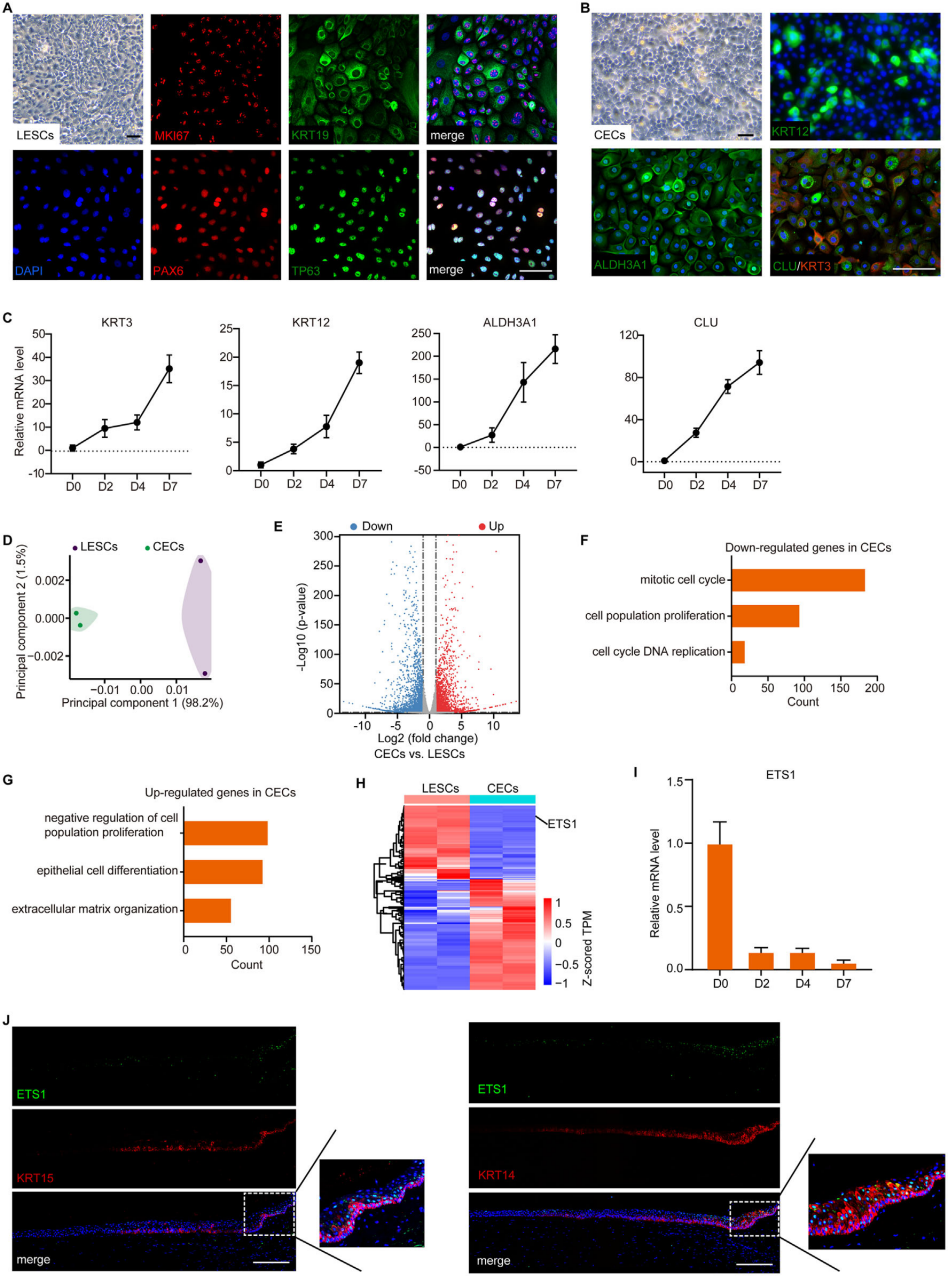
RNA-seq analysis identified ETS1 as an LESC-specific transcription factor. **(A)** Phase contrast image and immunofluorescence staining of primary LESCs for the indicated marker genes. *Scale bar:* 100 µm. **(B)** Representative phase contrast photographs of differentiated CECs that were induced in KSFM containing calcium chloride for seven days. Immunofluorescence staining for KRT3, KRT12, CLU, and ALDH3A1 in differentiated CECs. *Scale bar:* 100 µm. **(C)** QPCR analysis of gene expression changes in CEC markers during CEC differentiation. Data are presented as mean ± SE (n = 3). **(D)** Principal component analysis of transcriptome data of LESCs and CECs. **(E)** Volcano plot of differentially expressed genes between LESCs and CECs. The significantly differentially expressed genes (fold change ≥ 2 and *P* value < 0.05) are shown in *red* (upregulated genes) or *blue* (downregulated genes). **(F, G)** GO analysis of genes that were downregulated **(F)** or upregulated **(G)** in CECs. **(H)** Heatmap showing the differentially expressed transcription factors between LESCs and CECs. **(I)** QPCR analysis of gene expression changes for *ETS1* during differentiation. Data are presented as mean ± SE (n = 3). **(J)** Immunofluorescence staining for ETS1, KRT14, and KRT15 in normal human cornea. *Scale bar:* 100 µm.

We isolated and cultured human primary LESCs in vitro. The LESCs with high expansion ability were identified by the defined markers MKI67, KRT19, TP63, and PAX6 (Fig. 1A). Defined KSFM-containing insulin, epidermal growth factor, FGF, and a high concentration of calcium chloride is widely used for keratinocyte differentiation.^11^^,^^30^ We found that human LESCs treated with this differentiation medium for seven days showed extensive expression of CEC markers (KRT3, KRT12,^31^ CLU,^32^^,^^33^ and ALDH3A1^34^^–^^36^), indicative of a robust terminal differentiation (Fig. 1B). The expression of these CEC marker genes gradually increased during differentiation (Fig. 1C). Therefore, we used this protocol to differentiate LESCs into mature CECs in vitro. Then, RNA-seq was performed to generate genome-wide gene expression profiles for LESCs and CECs. Principal component analysis showed that the gene expression pattern was distinct between LESCs and CECs (Fig. 1D). Differential gene expression analysis showed that 1873 genes were downregulated during differentiation and that 1911 genes were upregulated (Fig. 1E). GO analysis showed that the downregulated genes in CECs were associated with mitotic cell cycle and cell proliferation (Fig. 1F), whereas the upregulated genes were linked to suppression of cell proliferation, epithelial cell differentiation, and extracellular matrix organization (Fig. 1G). Among the differentially expressed transcription factors, ETS1 exhibited a higher expression level in LESCs than in CECs (Fig. 1H). qPCR analysis also verified that the expression of *ETS1* dramatically decreased on differentiation (Fig. 1I). As expected, ETS1 was primarily expressed in the suprabasal layer of the limbal epithelium and was extremely weak in the central corneal epithelium (Fig. 1J). Of note, some of the KRT14/KRT15-postive LESCs in the basal layer of the limbal epithelium also showed the expression of ETS1 (Fig. 1J), suggesting that ETS1 is expressed in the LESCs.

### ETS1 Promoted LESC Proliferation and Inhibited Its Differentiation

**Figure 2.**
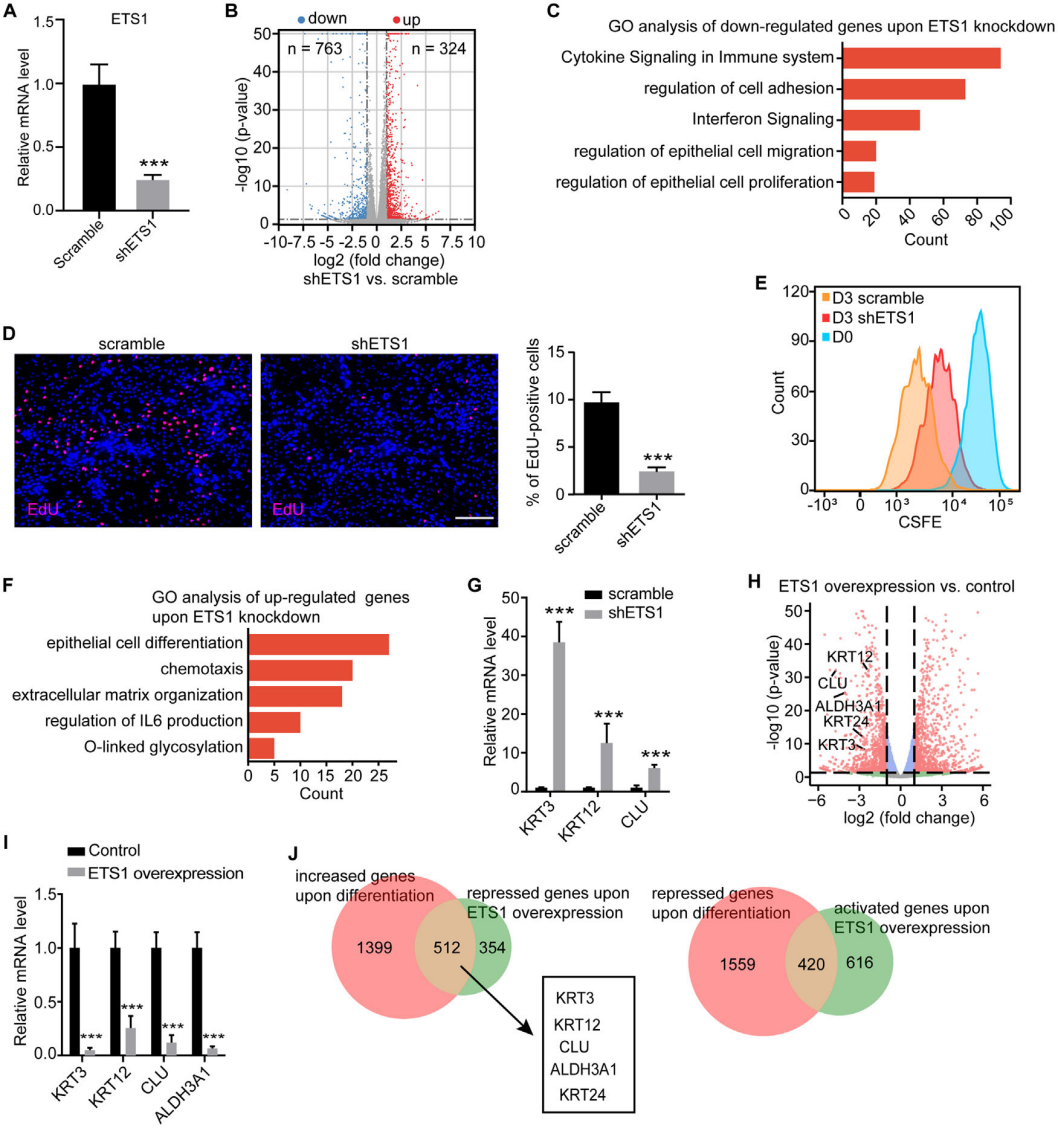
ETS1 promoted LESC proliferation and inhibited its differentiation. **(A)** QPCR analysis of the knockdown efficiency of *ETS1*. Data are presented as mean ± SE (n = 3, ***P < 0.001). **(B)** Volcano plot showing gene expression changes on *ETS1* knockdown. **(C)** GO analysis of genes that were downregulated on *ETS1* knockdown. **(D)** EdU staining (*left*) and quantifications of EdU-positive cells (*right*) in shETS1-treated and scrambled shRNA-treated LESCs. *Scale bar:* 200 µm. Data are presented as mean ± SE (n = 3, ***P < 0.001). **(E)** Flow cytometry analysis of CFSE labeling in the indicated groups. D0 represents the starting point of original fluorescence intensity. The scramble and shETS1 groups were analyzed at day 3 after CFSE labeling. **(F)** GO analysis of genes that were upregulated on *ETS1* knockdown. **(G)** QPCR analysis of CEC marker gene expression in shETS1-treated and scrambled shRNA-treated LESCs. Data are presented as mean ± SE (n = 3, ***P < 0.001). **(H)** Volcano plot showing gene expression changes induced by *ETS1* overexpression at day 7 after differentiation. **(I)** QPCR analysis of the expression levels of CEC markers at day 7 after differentiation in *ETS1* overexpression and control groups. Data are presented as mean ± SE (n = 3, ***P < 0.001). **(J)** Venn diagram showing overlapping genes between upregulated genes on differentiation and downregulated genes on *ETS1* overexpression and between downregulated genes on differentiation and upregulated genes on *ETS1* overexpression.

Given that ETS1 was expressed in LESCs, we next explored its function in LESCs. We knocked down *ETS1* in LESCs using shRNAs and then performed RNA-seq analysis (Figs. 2A, 2B). A cohort of differentially expressed genes, including 710 downregulated and 282 upregulated genes, were identified (Fig. 2B). GO analysis showed that the genes downregulated on *ETS1* knockdown were enriched for the biological processes associated with proliferation and immunological responses (Fig. 2C). We then used EdU to assess the effect of ETS1 on LESC proliferation. The *ETS1* knockdown group showed a much lower percentage of EdU-positive cells than LESCs treated with scrambled shRNA (Fig. 2D). CFSE is a protein-labeling fluorescent tracer, the fluorescence intensity of which is reduced by half after each cell division. Flow cytometry analysis showed that the CFSE fluorescence intensity of *ETS1*-depleted LESCs was significantly higher than that of the control group on day 3 after CFSE labeling (Fig. 2E). These results indicated that loss of *ETS1* inhibited LESC proliferation.

In contrast, *ETS1* knockdown increased the expression of genes that regulate epithelial cell differentiation and extracellular matrix organization (Fig. 2F). Furthermore, we found that *ETS1* depletion activated the CEC markers, KRT3, KRT12, and CLU (Fig. 2G). To further verify the potential role of ETS1 in LESC differentiation, we overexpressed *ETS1* during CEC differentiation. Both RNA-seq and qPCR analyses indicated that *ETS1* overexpression repressed the expression of CEC markers, *KRT3*, *KRT12*, *KRT24*, *CLU*, and *ALDH3A1* (Figs. 2H, 2I). In addition, approximately 59% (512/866) of the downregulated genes induced by *ETS1* overexpression were CEC-associated genes. Approximately 41% (420/1036) of the genes upregulated on *ETS1* overexpression were LESC-associated genes (Fig. 2J). Collectively, these results showed that ETS1 promoted LESC proliferation and inhibited its differentiation.

### ETS1 Regulated Downstream Genes Through Promoters or Enhancers

**Figure 3.**
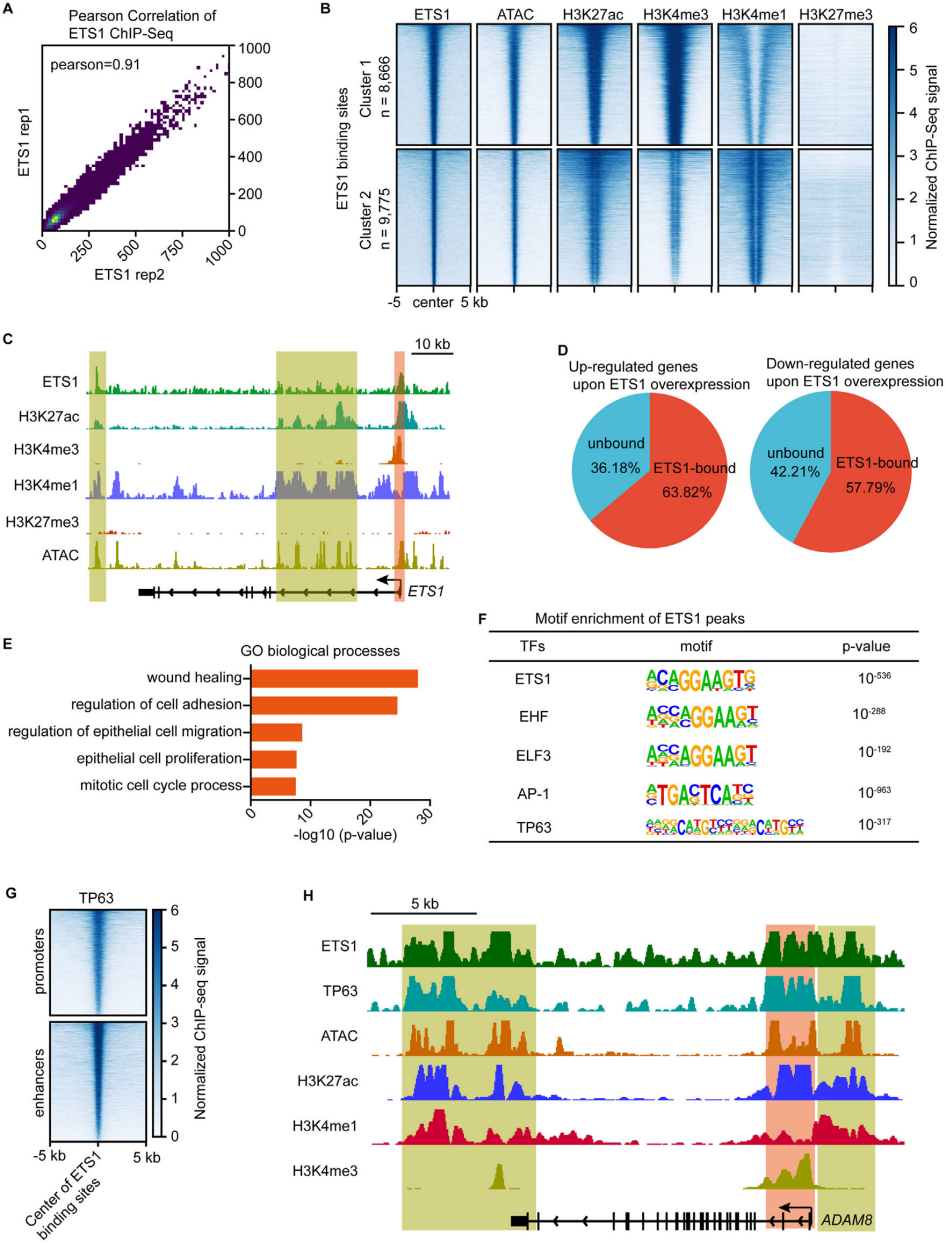
ETS1 regulated target genes via promoters and/or enhancers. **(A)** Pearson's correlation coefficient analysis of two independent biological replicates of ETS1 ChIP-seq samples. **(B)** Heatmaps showing the indicated ChIP-seq and ATAC-seq signals at ETS1-binding sites. **(C)** Genome browser tracks for the indicated ChIP-seq and ATAC signals across *ETS1* locus. **(D)** Pie charts showing the percentage of upregulated or downregulated genes on ETS1 overexpression that were bound by ETS1. **(E)** GO analysis of ETS1-bound genes. **(F)** Motif enrichment for ETS1 peaks. **(G)** Heatmaps showing the ChIP-seq signal of TP63 at ETS1-binding sites in LESCs. (H) Genome browser tracks for the indicated ChIP-seq and ATAC signals across *ADAM8* locus.

To further elucidate the potential mechanism of ETS1-dependent transcriptional regulation, the genome-wide binding profile of ETS1 was mapped using ChIP-seq. Pearson's correlation coefficient analysis showed a high degree of similarity between two independent biological replicates (Fig. 3A). In general, transcription factors activate or repress gene transcription by binding to *cis*-regulatory elements that are marked by defined histone modifications. The ChIP-seq data for active (H3K27ac, H3K4me1, and H3K4me3) and repressive (H3K27me3) histone modifications in LESCs have been generated in our previous publication.^20^ We also previously profiled the chromatin accessibility landscape of LESCs by ATAC-seq.^20^ Combined with these epigenetic maps, we showed that the binding pattern of ETS1 paralleled that of ATAC and H3K27ac (Fig. 3B), indicative of an active status. The ETS1-binding sites were clustered into two groups: cluster 1 (8666 peaks) represented active promoters with H3K27ac/H3K4me3 positivity and H3K4me1 negativity; cluster 2 (9775 peaks) were active enhancers defined by highH3K27ac and H3K4me1 enrichment. Both clusters were open and lacked the repressive H3K27me3 signal^37^ (Fig. 3B).

We found that the promoter of ETS1 was active and enriched for strong H3K27ac, H3K4me3, and ATAC signals (Fig. 3C). Multiple active enhancers that regulated ETS1 were also identified based on the enrichment of H3K27ac, H3K4me1, and ATAC peaks (Fig. 3C). Intriguingly, ETS1 bound to its own promoter and a distal enhancer (Fig. 3C), indicating a self-regulation. Remarkably, the promoters or enhancers of over half of the differentially expressed genes induced by ETS1 overexpression were directly occupied by ETS1 (Fig. 3D). Consistent with the results of RNA-seq analysis, the target genes of ETS1 were enriched for GO terms associated with proliferation and cell cycle (Fig. 3E). These observations demonstrated that ETS1 regulated downstream genes through promoters and/or enhancers. Key TFs often cooperate with multiple regulators to control gene transcription. We performed transcription factor motif enrichment analysis for ETS1 peaks using the HOMER algorithm. We found that ETS1-binding sites were significantly enriched for motifs of well-known important corneal epithelial regulators, including EHF,^38^ ELF3,^39^ AP-1^40^^,^^41^ and TP63^42^ (Fig. 3F). Combined with the TP63 ChIP-seq data generated in our previous document,^25^ we found that the ETS1-binding sites including promoters and enhancers were also co-occupied by TP63 in LESCs (Fig. 3G), as exemplified across *ADAM8* locus (Fig. 3H). The co-location of ETS1 and TP63 across the genome implied that ETS1 might coordinate with TP63 to maintain LESC functions.

### HMGA2 as a Downstream Effector of ETS1 Regulated LESC Proliferation and Differentiation

**Figure 4.**
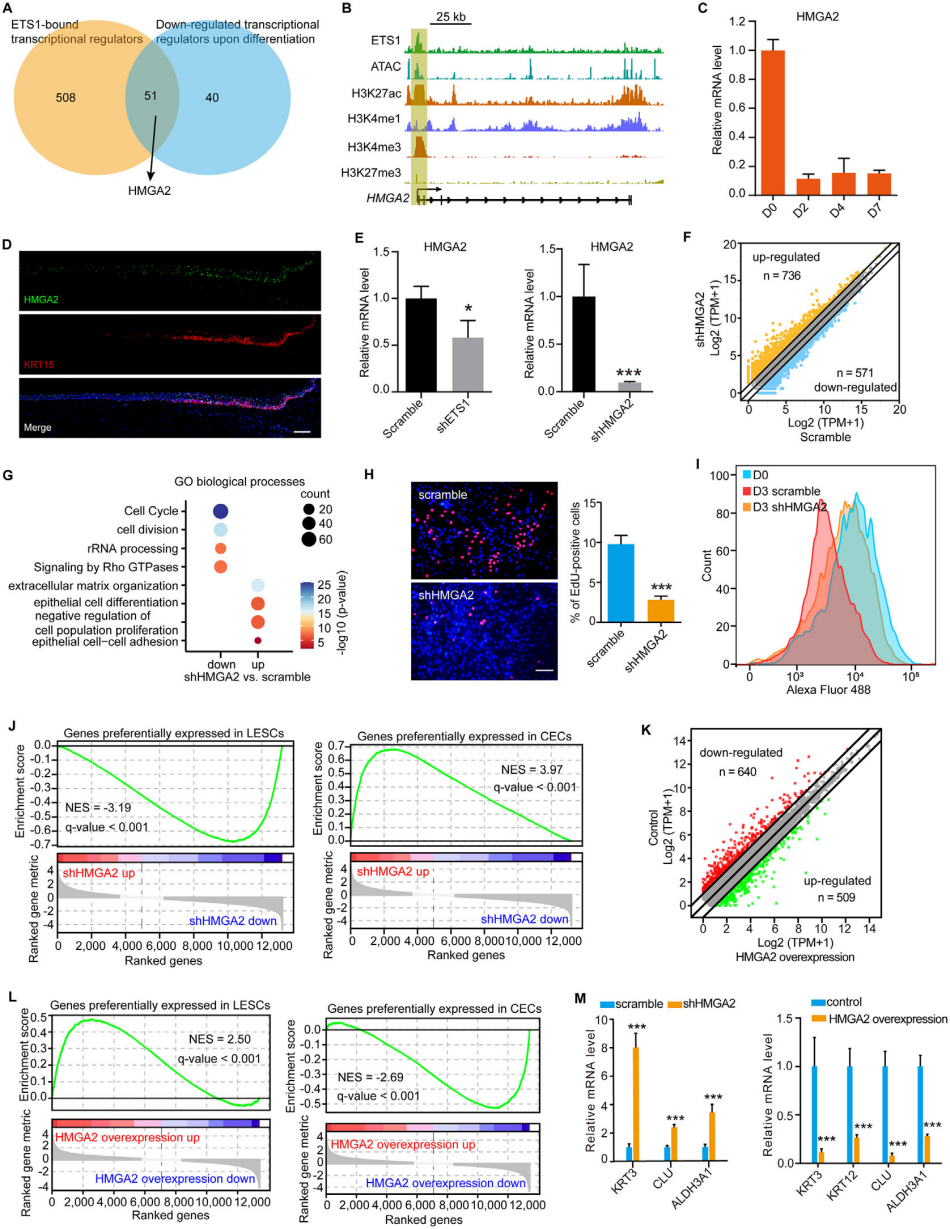
HMGA2 was required for the maintenance of proliferation and undifferentiated state of LESCs. **(A)** Venn diagram showing overlapping between ETS1-bound transcriptional regulators and downregulated transcriptional regulators on differentiation. **(B)** Genome browser tracks for the indicated ChIP-seq and ATAC signals across *HMGA2* locus. **(C)** QPCR analysis of change in *HMGA2* expression during CEC differentiation. Data are presented as mean ± SE (n = 3). **(D)** Immunofluorescence staining for HMGA2 and KRT15 in normal human corneal tissue. *Scale bar:* 100 µm. **(E)** QPCR analysis of *HMGA2* expression in shETS1-treated and scrambled shRNA-treated LESCs (*left*). QPCR analysis of the knockdown efficiency of *HMGA2* (*right*). Data are presented as mean ± SE (n = 3, *P < 0.05, ***P < 0.001). **(F)** The differentially expressed genes between shHMGA2-treated and scrambled shRNA-treated LESCs. **(G)** GO analysis of genes that were downregulated or upregulated on *HMGA2* knockdown. **(H)** EdU staining (*left*) and quantifications of EdU-positive cells (*right*) in shHMGA2-treated and scrambled shRNA-treated LESCs. *Scale bar:* 100 µm. Data are presented as mean ± SE (n = 3, ***P < 0.001). **(I)** Flow cytometry analysis of CFSE labeling in the indicated groups. D0 represents the starting point of original fluorescence intensity. The scramble and shHMGA2 groups were analyzed at day 3 after CFSE labeling. **(J)** GSEA of gene sets that were preferentially expressed in LESCs and CECs when *HMGA2* was knocked down. NES, normalized enrichment score. **(K)** The differentially expressed genes generated by *HMGA2* overexpression after differentiation. **(L)** GSEA of gene sets that were preferentially expressed in LESCs and CECs when *HMGA2* was overexpressed. **(M)** QPCR analysis of CEC marker gene expressions in shHMGA2-treated and scrambled shRNA-treated LESCs (*left*). QPCR analysis of the expression levels of CEC markers at day 7 after differentiation when *HMGA2* was overexpressed (*right*). Data are presented as mean ± SE (n = 3, ***P < 0.001).

To identify the downstream effectors of ETS1, we obtained the transcriptional regulators that were bound by ETS1 in LESCs. By overlapping them with the transcriptional regulators that were downregulated on differentiation, we focused on HMGA2 (Fig. 4A), which is a chromatin regulator that involves transcriptional regulation.^43^ We found that the promoter of *HMGA2* was significantly enriched for H3K27ac, H3K4me3, and ATAC signals in LESCs (Fig. 4B), indicative of a highly activated state. Importantly, this active promoter was bound by ETS1 (Fig. 4B). The expression of *HMGA2* was dramatically decreased after differentiation (Fig. 4C), which was consistent with the expression pattern of *ETS1* (Fig. 1H). Further in vivo experiment also showed that HMGA2 was preferentially expressed in the limbal epithelium especially in the basal layer (Fig. 4D). Knockdown of *ETS1* inhibited the expression of *HMGA2* (Fig. 4E). These observations suggested that HMGA2 may be a potential downstream effector of ETS1.

To explore the function of HMGA2 in LESCs, we knocked down *HMGA2* and identified a cohort of differentially expressed genes, including 736 upregulated and 571 downregulated genes (Fig. 4F). GO analysis showed that the downregulated genes were associated with cell cycle (Fig. 4G). The upregulated genes were linked to epithelial differentiation and negative regulation of cell proliferation (Fig. 4G). Both the EdU and CFSE staining experiments showed that knockdown of *HMGA2* significantly inhibited LESC proliferation (Figs. 4H, 4I). In addition, we found that loss of *HMGA2* decreased the expression of genes that were preferentially expressed in LESCs (Fig. 4J). In contrast, the genes with a higher expression level in CECs than in LESCs were upregulated when *HMGA2* was knocked down (Fig. 4J). Furthermore, we overexpressed *HMGA2* when LESCs were induced to differentiate, identifying 640 downregulated and 509 upregulated genes (Fig. 4K). The LESC-associated gene set was activated and the CEC-associated gene set was repressed when *HMGA2* was overexpressed during differentiation (Fig. 4L). As expected, the expression of CEC markers was activated on *HMGA2* knockdown in LESCs and was inhibited on *HMGA2* overexpression during differentiation (Fig. 4M). We found that 92 downregulated and 119 upregulated genes were overlapped between *ETS1*-depleted and *HMGA2*-depleted LESCs (Supplementary Fig. S1). Approximately half of the differentially expressed genes induced by *HMGA2* overexpression showed the consistent alteration when *ETS1* was overexpressed (Supplementary Fig. S1). These results indicated that HMGA2, which acted as a downstream effector of ETS1, promoted LESC proliferation and inhibited its differentiation.

## Discussion

The structural integrity and transparency of the non-keratinized stratified squamous corneal epithelium are essential for corneal barrier and visual function. Located in the basal layer of the limbal epithelium, LESCs play important roles in the self-renewal and differentiation of the corneal epithelium.^44^^,^^45^ During corneal epithelium homeostasis and regeneration, the proliferation and differentiation of LESCs are indispensable.^5^ On injury, adjacent corneal epithelial cells immediately flatten and migrate to seal the wound area. Once the integrity of the corneal epithelium is re-established, LESCs proliferate and differentiate into CECs to repopulate the wound area.^4^ Therefore it is important to understand the mechanisms whereby LESC proliferation and differentiation are controlled.

Emerging evidences demonstrate that transcription factors play important roles in cell proliferation. Various transcription factors that regulate LESC proliferation have also been identified. TP63 is a stratified epithelial-specific transcription factor that is required for epithelial stem cell proliferation and epithelial stratification.^46^^–^^48^ KLF4,^49^ KLF5,^50^ and CEBPD^51^ are also three key regulators that promote cell cycle progression in LESCs. their loss-of-function mutations result in dysregulated corneal epithelial homeostasis. Here, we identified ETS1 as a novel key regulator expressed in the limbal epithelium that maintains proliferative capacity of LESCs. We found that *ETS1* knockdown inhibited cell proliferation but activated the differentiation program in LESCs, which is consistent with the results observed in skin keratinocytes.^16^^,^^17^ In addition, it has been established that ETS1 can promote cell proliferation in squamous cell carcinoma, indicating functional conversation of ETS1.^19^ The co-occupancy of ETS1 and TP63 at the cis-regulatory elements suggested that ETS1 might coordinate with TP63 to control LESC function.

In vivo observation suggested that ETS1 was expressed in the KRT14/KRT15-positive LESCs residing in the basal cell layer of the limbal epithelium. Despite the important role of ETS1 in proliferation of LESCs, ETS1 was also expressed in the suprabasal layer of the limbal epithelium. As limbal suprabasal epithelial cells do not proliferate, the function of ETS1 in the limbal suprabasal epithelial cells might not be associated with proliferation. ETS1 is known to be involved in multiple biological functions in normal cells, including regulating angiogenesis^52^ and immunity response.^53^ We speculated that the function of ETS1 in the limbal suprabasal epithelial cells might be different from that in LESCs. The role of ETS1 in the limbal suprabasal epithelium need to be further explored in the future.

HMGA2 is a chromatin architectural protein and can regulate gene transcription by interacting with the transcription factors or epigenetic regulators.^43^ HMGA2 protein is highly expressed in embryonic stem cells and in proliferative stem cells during embryonic development.^54^ HMGA2 expression is also observed in some adult stem cells.^55^ We showed that HMGA2 was preferentially expressed in the human limbal epithelium, including KRT15-positive LESCs residing in the basal layer. Consisting with the results of the in vitro differentiation, HMGA2 expression decreased progressively from the limbal epithelium to the central corneal epithelium. Numerous studies have suggested that HMGA2 can promote self-renewal and stemness maintenance of some adult stem cells.^55^ We found that HMGA2 promoted LESC proliferation and inhibited the differentiation, which was consistent with the results observed in other adult stem cells. We also showed that ETS1 and HMGA2 shared the similar function, and ETS1 activated HMGA2 expression through direct binding to its promoter in LESCs. Taken together, we proposed a fundamental molecular mechanism that regulates LESC proliferation and differentiation.

## Supplementary Material

Supplement 1

Supplement 2
