# Supplementary material for: ETS1–HMGA2 Axis Promotes Human Limbal Epithelial Stem Cell Proliferation
Source: Invest Ophthalmol Vis Sci. 2023 Jan 18;64(1):12. doi: 10.1167/iovs.64.1.12 (PMC9855287; doi:10.1167/iovs.64.1.12)
Supplement: Supplement 1 [file iovs-64-1-12_s001.pdf]

Supplementary Table1. Information of antibodies for Immunofluorescence

Antibody	Dilution	Company and Cat number
anti-ETS1	1:500	Thermofisher, Cat# PA5-38270
anti-KRT3	1:200	Abcam, Cat# ab68260
anti-KRT12	1:200	Abcam, Cat# ab124975
anti-CLU	1:100	Proteintech, Cat# 12289-1-AP
anti-ALDH3A1	1:300	GeneTex, Cat# GTX30042
anti-KRT14	1:200	Thermofisher, Cat# MA5-11599
anti-Rabbit IgG (Alexa Fluor 488)	1:1000	Cell Signaling Technology, Cat# 4412S
anti-mouse-IgG (Alexa Fluor 594)	1:1000	Cell Signaling Technology, Cat# 8890S
anti-TP63	1:500	GeneTex, Cat# GTX102425
anti-PAX6	1:500	Sigma, Cat# AMAB91372
anti-Cytokeratin 19	1:500	Abcam, Cat# ab52625
anti-Ki67	1:1000	Cell Signaling Technology, Cat# 9449
Anti-KRT15	1:200	Thermofisher, Cat# MA5-11344

Supplementary Table2. Information of shRNAs

scramble	CAACAAGATGAAGAGCACCAA
shETS1-1	GACCGTGCTGACCTCAATAAG
shETS1-2	GTGCAGATGTCCCACTATTAA
shHMGA2-1	GGCCAAGAGGCAGACCTAGGA
shHMGA2-2	CCGAATTGGGTTTAGTCAATC
