# Supplementary material for: ETS1–HMGA2 Axis Promotes Human Limbal Epithelial Stem Cell Proliferation
Source: Invest Ophthalmol Vis Sci. 2023 Jan 18;64(1):12. doi: 10.1167/iovs.64.1.12 (PMC9855287; doi:10.1167/iovs.64.1.12)
Supplement: Supplement 2 [file iovs-64-1-12_s002.pdf]

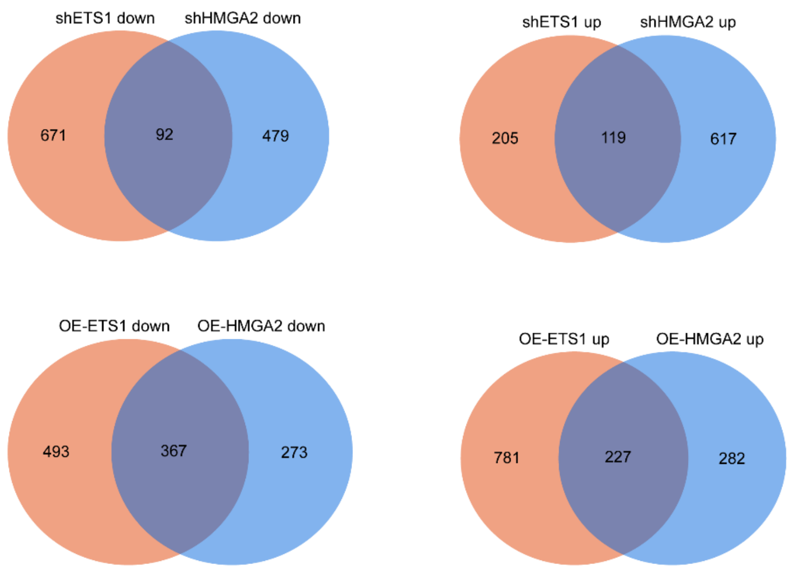

Supplementary figure 1. The overlapping between genes that were altered when *ETS1* or *HMGA2* is modulated. OE, overexpression.
